# Spatial analysis of recurrent glioblastoma reveals perivascular niche organization

**DOI:** 10.1172/jci.insight.179853

**Published:** 2024-05-23

**Authors:** Ugoma Onubogu, Chandler D. Gatenbee, Sandhya Prabhakaran, Kelsey L. Wolfe, Benjamin Oakes, Roberto Salatino, Rachael Vaubel, Oszkar Szentirmai, Alexander R.A. Anderson, Michalina Janiszewska

**Affiliations:** 1 The Skaggs Graduate School of Chemical and Biological Science, The Scripps Research Institute, La Jolla, California, USA.; 2 Department of Molecular Medicine, The Herbert Wertheim UF Scripps Institute for Biomedical Innovation & Technology, Jupiter, Florida, USA.; 3 Department of Mathematical Oncology, H. Lee Moffitt Cancer Center and Research Institute, Tampa, Florida, USA.; 4 Department of Laboratory Medicine and Pathology, Mayo Clinic Rochester, Rochester, Minnesota, USA.; 5 Center for Neurological Surgery and Neuroscience, Cleveland Clinic Martin Health, Port St. Lucie, Florida, USA.

**Keywords:** Oncology, Brain cancer, Macrophages, Molecular pathology

## Abstract

Tumor evolution is driven by genetic variation; however, it is the tumor microenvironment (TME) that provides the selective pressure contributing to evolution in cancer. Despite high histopathological heterogeneity within glioblastoma (GBM), the most aggressive brain tumor, the interactions between the genetically distinct GBM cells and the surrounding TME are not fully understood. To address this, we analyzed matched primary and recurrent GBM archival tumor tissues with imaging-based techniques aimed to simultaneously evaluate tumor tissues for the presence of hypoxic, angiogenic, and inflammatory niches, extracellular matrix (ECM) organization, *TERT* promoter mutational status, and several oncogenic amplifications on the same slide and location. We found that the relationships between genetic and TME diversity are different in primary and matched recurrent tumors. Interestingly, the texture of the ECM, identified by label-free reflectance imaging, was predictive of single-cell genetic traits present in the tissue. Moreover, reflectance of ECM revealed structured organization of the perivascular niche in recurrent GBM, enriched in immunosuppressive macrophages. Single-cell spatial transcriptomics further confirmed the presence of the niche-specific macrophage populations and identified interactions between endothelial cells, perivascular fibroblasts, and immunosuppressive macrophages. Our results underscore the importance of GBM tissue organization in tumor evolution and highlight genetic and spatial dependencies.

## Abstract 2

Comparison of primary and recurrent glioblastoma reveals collagen-rich niches around blood vessels that provide a unique environment that contributes to immune suppression.

## Graphical abstract

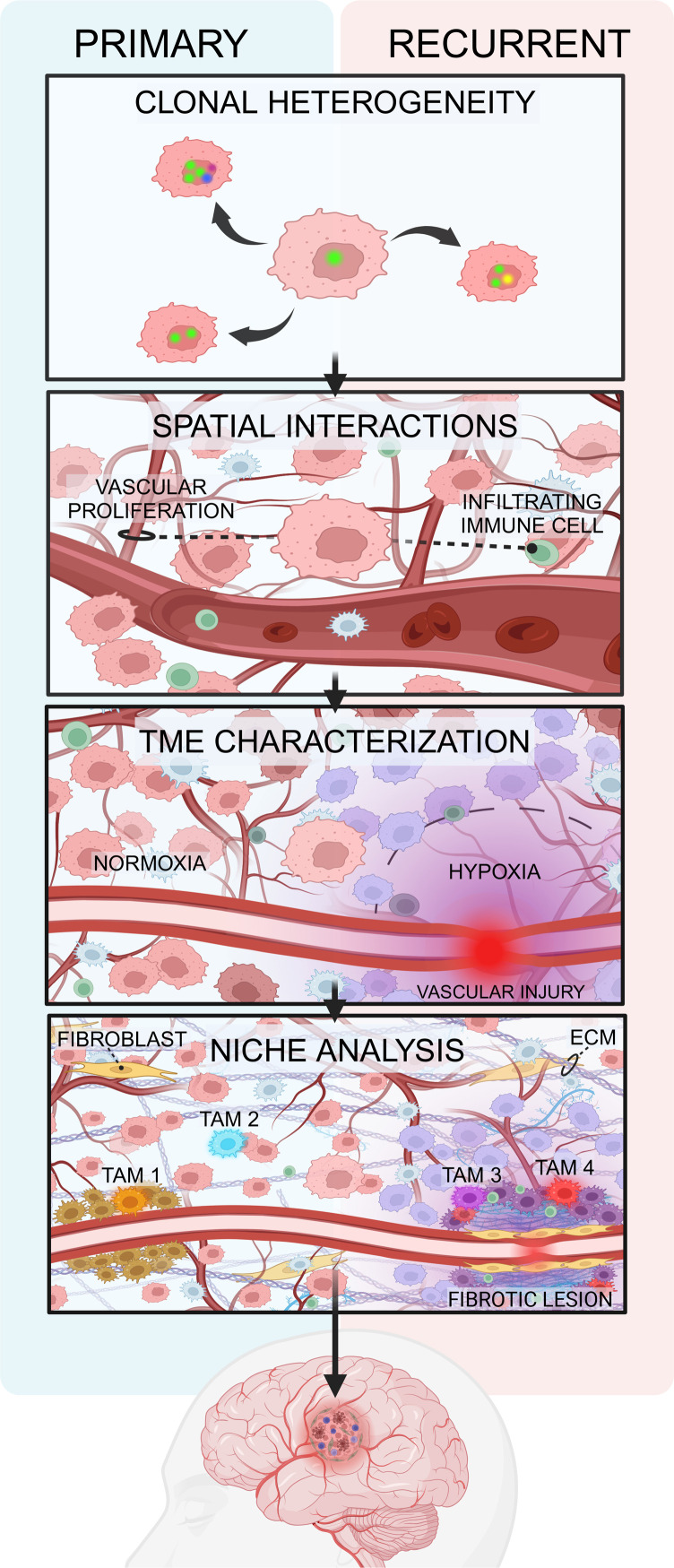

## Introduction

Diversity of histopathological features in tumor tissue is one of the hallmarks of glioblastoma (GBM) (1). This aggressive brain tumor is characterized by the presence of pseudopalisading necrotic regions intermixed with microvascular proliferation areas in a single biopsy of the tumor (1). GBM remains one of the biggest challenges in medical oncology, as virtually all tumors relapse despite intensive treatment with surgery, chemotherapy, and radiation. The lack of treatments based on molecular targets is largely due to extreme intratumor heterogeneity of GBM, reflected at all levels, from histology to single-cell transcriptomic, epigenetic, and genetic heterogeneity (2). Given that the main histological features co-occurring in GBM constitute opposing microenvironments, hypoxic perinecrotic areas and highly vascularized oxygen-rich niches, it is likely that they contribute to the selection of distinct traits in tumor cells. Thus, studying the role of the tumor microenvironment (TME) in shaping diverse landscapes composed of genetically distinct subpopulations of cells is key to understanding the dynamics of GBM evolution.

Single-cell transcriptomics enabled the characterization of distinct GBM cell types coexisting in each tumor. The most widely adopted classification of GBM cellular heterogeneity links specific copy number changes to cellular states mirroring normal early brain development (2). Amplifications of *EGFR*, *CDK4*, and *PDGFRA* are associated with astrocyte-like (AC-like), neural progenitor cell–like (NPC-like), and oligodendrocytic precursor cell–like (OPC-like) states, respectively. Recent advances in spatial profiling facilitated the investigation of the architecture of GBM tissues and allowed for the identification of several patterns of spatial organization in these tumors (3, 4). However, the link between genetically defined subpopulations and their microenvironment and changes in these relationships that may drive recurrence remain to be further investigated.

In this study, we explore the spatial relationships between genetic heterogeneity and diversity of the TME at the single-cell level in a cohort of matched primary and recurrent human GBM tissues. Our multiplexed confocal imaging–based approach identified links between the texture of extracellular matrix (ECM) components, genetically distinct subpopulations of GBM cells, and cells of the TME. Moreover, we also discovered a differential ECM organization in perivascular niches in recurrent GBM, enriched in immunosuppressive macrophages possessing unique transcriptional features. The specific localization of these immunosuppressive cells could provide a novel axis for alleviating immune inhibition contributing to poor survival in GBM patients.

## Results

### Multiplexed imaging uncovers correlations between genotype and TME.

**Figure 1:**
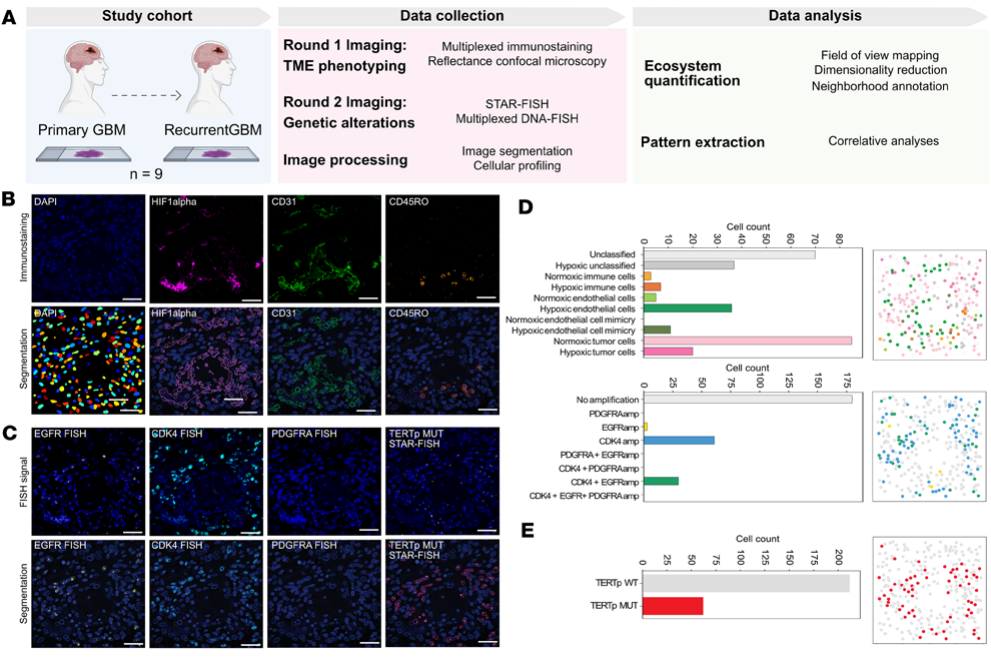
Immunogenotyping of primary and recurrent archival GBM samples. (**A**) Study outline. (**B**) Top panels: Representative images of immunofluorescent staining for markers of hypoxia (HIF1α), endothelial cells (CD31), and immune cells (CD45RO). Bottom panels: Image segmentation. (**C**) Top panels: Representative FISH images for *CDK4*, *EGFR*, and *PDGFRA* and STAR-FISH for *TERT* promoter (*TERT*p) mutation, respectively. Bottom panels: Image segmentation. Scale bars: 40 μm (**B** and **C**). (**D**) Left panels: Quantification of cell frequency based on phenotypes (top) and genotypes (bottom) in representative images in **B** and **C**. Right panels: Spatial distribution of cells classified into distinct phenotypes and genotypes corresponding to the genotype panels on the left. (**E**) Top panel: Quantification of cell frequency based on *TERT*p mutation status in representative images in **B** and **C**. Bottom panel: Spatial distribution of cells classified into *TERT*p WT and MUT.

In our previous study, we showed that the single-cell mosaicism of genetic amplifications in GBM correlates with immune infiltration (5). To test whether we could identify features of the TME that drive the selection of specific amplifications, we conducted a multiplexed imaging study on formalin-fixed, paraffin-embedded (FFPE) samples from a cohort of 9 matched primary and recurrent GBM cases (Figure 1A and Supplemental Table 1; supplemental material available online with this article; https://doi.org/10.1172/jci.insight.179853DS1). The imaging was performed in 2 consecutive rounds on the same tissue, using modified CyCIF and STAR-FISH protocols (5–7) (see Methods for details). First-round imaging was aimed to acquire information about the TME state by assessing the presence of blood vessels (CD31), active infiltrating immune cells (CD45RO), and hypoxia (HIF1α) (Figure 1B). The next layer of imaging was focused on genetic features, including amplifications of *EGFR*, *CDK4*, and *PDGFRA*, and hotspot mutation in the *TERT* promoter (*TERT*p) (Figure 1C). The 3 amplifications were previously associated with distinct transcriptional states in GBM, namely NPC-like state with *CDK4* amplification*,* AC-like state with *EGFR* amplification, and OPC-like state with *PDGFRA* amplification (2). Nuclear staining channels from the first and second rounds of imaging were overlaid to ensure no loss of cells occurred (loss of a single cell occurred in 9 imaged regions and between 2 and 6 cells in 3 imaged regions; total count of lost cells was 22 out of 20,205; Supplemental Figure 1). Image segmentation and quantification of both the nuclear and TME-related staining allowed us to classify each nucleus as belonging to a tumor cell with or without one or a combination of the 3 amplifications, a tumor cell with *TERT*p mutation, an immune cell, or an endothelial cell (EC) (Figure 1, B–E, and Supplemental Table 2). GBM cells can mimic ECs and pericytes (8, 9). Indeed, we identified 958 cells characterized by tumor-specific genetic markers and expressing the EC/pericyte marker CD31. The hypoxia marker, nuclear HIF1α, can be expressed both by tumor cells and immune cells in the TME and our analysis identifies both populations. Of note, cells harboring normal *CDK4*, *EGFR*, and *PDGFRA* copy number, no mutation, and no immune or endothelial markers, which may represent normal cells such as oligodendrocytes, neurons, or astrocytes, as well as tumor cells negative for our tumor markers, were excluded from quantitative analyses and only considered in spatial analyses for accurate measurement of regional cell density.

**Figure 2:**
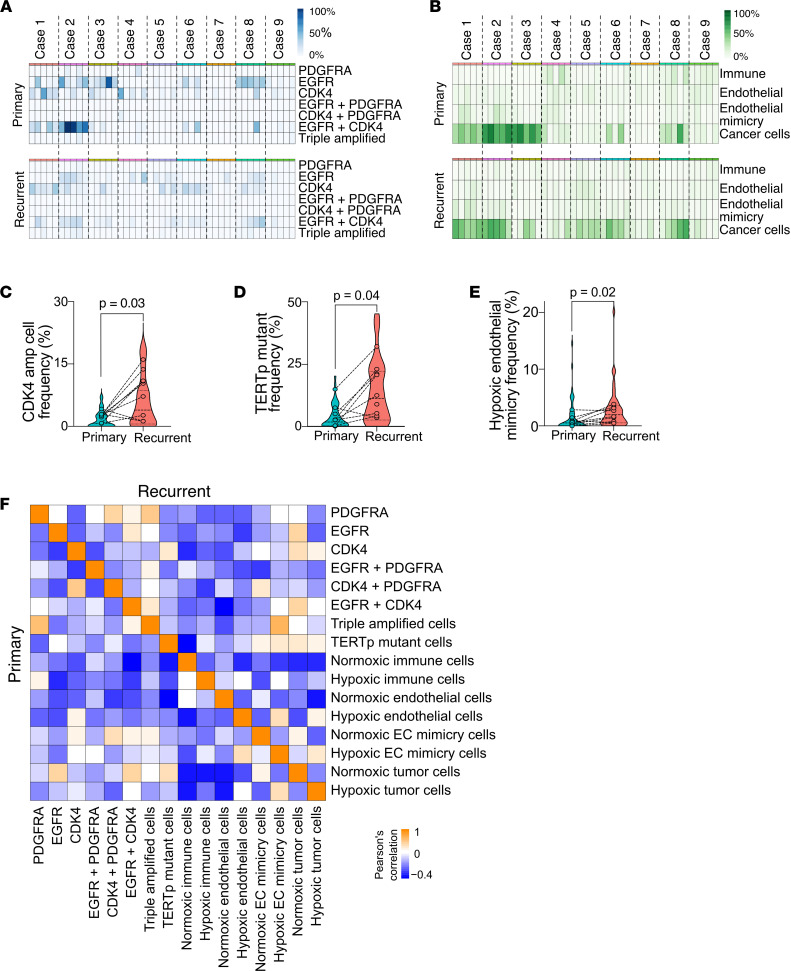
Differential cell frequencies and correlations between genotypes and phenotypes in primary and recurrent tumors. (**A**) Frequency of cells with distinct genotypes in primary and recurrent GBM for each of 5 imaged tumor regions per case (*n* = 9 matched cases). (**B**) Frequency of cells with distinct phenotypes in primary and recurrent GBM for each of 5 imaged tumor regions per case (*n* = 9 matched cases). (**C**) Frequency of cells with *CDK4* amplification in matched primary and recurrent samples. Data points represent case average frequency after ROUT outlier removal. Dotted line: matched primary and recurrent cases. Violin plot shows mean and quartiles. *P* value of 2-tailed paired Wilcoxon’s ranked test is shown. (**D**) Frequency of cells with *TERT* promoter (*TERT*p) mutation in matched primary and recurrent samples. Data points represent case average frequency after ROUT outlier removal. Dotted line: matched primary and recurrent cases. Violin plot shows mean and quartiles. *P* value of 2-tailed paired Wilcoxon’s ranked test is shown. (**E**) Frequency of cells with *TERT*p mutation in matched primary and recurrent samples. Data points represent case average frequency after ROUT outlier removal. Dotted line: matched primary and recurrent cases. Violin plot shows mean and quartiles. *P* value of 2-tailed paired Wilcoxon’s ranked test is shown. (**F**) Pearson’s correlation between the frequencies of cells with distinct genotypes and phenotypes.

First, we searched for changes in the frequencies of genotypes and phenotypes between matched primary and recurrent cases (Figure 2, A and B, and Supplemental Table 3). The frequency of cells with *CDK4* amplification and cells with *TERT*p mutation was higher in recurrent samples (Figure 2, C and D; for CDK4 comparisons; 2-tailed paired Wilcoxon’s ranked test, *P* = 0.03; for *TERT*p mutant comparisons: 2-tailed paired Wilcoxon’s ranked test, *P* = 0.04). Among the phenotypes we identified, hypoxic EC mimicry was more prevalent in recurrent samples (Figure 2E; 2-tailed paired Wilcoxon’s ranked test, *P* = 0.02). To account for overall diversity of each imaged field of view, we calculated the Shannon index of diversity (see Methods), capturing the evenness of distribution of cells among the different genotypes or phenotypes in each tumor (Supplemental Figure 2). These indices were significantly different between primary and recurrent samples, when amplification or *TERT*p mutation status was taken into consideration (2-tailed paired Wilcoxon’s ranked test, *P* = 0.0023 and *P* value = 0.0025, respectively). Thus, the distribution of cells harboring *TERT*p mutation within each tumor is more heterogeneous than the variation in hypoxia, vasculature, or immune infiltration.

Next, we calculated Pearson’s correlation between the frequencies of cells with distinct genotypes and phenotypes (Figure 2F). The most notable difference between primary and recurrent tumor samples was the association between hypoxic tumor cells adopting an EC phenotype (hypoxic EC mimicry) and a genotype consisting of all 3 amplifications co-occurring in the same cell. This correlation was significant, but weak in primary tumor samples, with *r* = 0.3 and *P* = 0.041, and strengthening in recurrence, with *r* = 0.73 and *P* = 0.9 × 10^–8^, suggesting that co-amplification of *EGFR*, *CDK4*, and *PDGFRA* may allow more tumor cells to adopt this hypoxia-driven perivascular phenotype.

Previously, we showed that the relative frequency of co-amplification of *EGFR* and *CDK4* at the single-cell level is linked with an immunosuppressive microenvironment in GBM (5). To test whether this association holds in recurrent tumors, we calculated the odds ratio (OR) for co-amplification of *EGFR* and *CDK4* for each tumor. The classification of our tumors into OR^high^ and OR^low^ revealed a significant difference in overall immune infiltration only in primary tumors, but not in the recurrent samples (Supplemental Figure 3). Thus, it is likely that these genetic drivers are more important in the early steps of establishing the immunosuppressive microenvironment.

**Figure 3:**
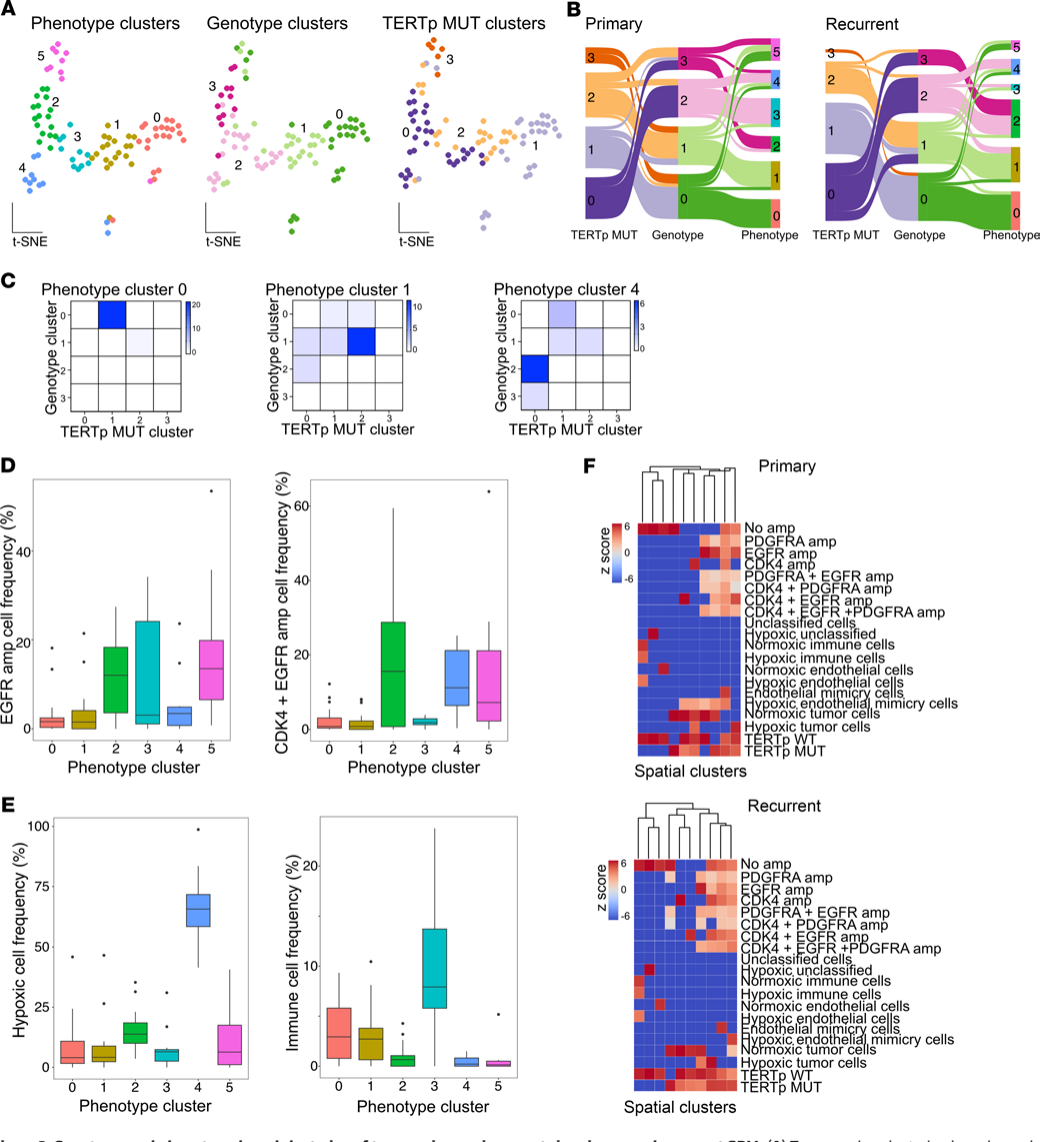
Genotype- and phenotype-based clustering of tumor microenvironments in primary and recurrent GBM. (**A**) Tumor region clustering based on cellular phenotypes, genotypes, and *TERT* promoter–mutant (*TERT*p-mutant) cells. Each point represents a tumor region. Numbers represent cluster identifier. (**B**) Connections between classification based on *TERT*p mutation, genotype, or phenotype clustering in primary and recurrent GBM. The width of each connection represents the number of tumor regions classified. (**C**) Contingency between the phenotypes and genotypes. Fisher’s exact test *P* values are: Cluster 0, *P* < 0.0001; Cluster 1, *P* < 0.0001; Cluster 4, *P* = 0.009. The color scale represents number of tumor regions classified. (**D**) Frequency of cells with *EGFR* amplification (left) and *CDK4/EGFR* co-amplification (right) across phenotype clusters. (**E**) Frequency of hypoxic (left) and immune cells (right) across phenotype clusters. The box-and-whisker plots in all bar graphs show the mean (midline) and 25th–75th (box) and 5th–95th (whiskers) percentiles. (**F**) Spatial distribution of cells with different genotypes and phenotypes. Columns in the heatmap represent spatial clusters determined based on XY coordinates of the cells.

To better understand the relationships between the genotypic and phenotypic diversity in our cohort, we next performed clustering analysis based on the frequencies of all cell types identified in each imaged region of interest. Clustering of individual images based on *TERT*p mutation frequencies revealed 4 classes of tumor areas. Similarly, amplification-based genotype clustering revealed 4 clusters of tumor areas, while phenotype frequencies divided the imaged tumor regions into 6 clusters (Figure 3, A and B). Most notable were the phenotype cluster 3, characterized by high frequency of immune cells, and cluster 4, with highest abundance of hypoxic cells (Supplemental Figure 4). Phenotype clusters 0 and 1 are both composed of intermediate frequencies of immune cells and ECs, but differ in overall cellularity (Supplemental Figure 4A). Interestingly, in addition to the highly hypoxic cluster 4, it was the more complex phenotypes of clusters 0 and 1 that were significantly associated with particular genotypes (Figure 3C). Tumor regions in phenotype cluster 0 are largely classified as genotype cluster 0 and mutation cluster 1, indicating a low frequency of all measured amplifications and depletion of *TERT*p-mutant cells despite overall high cellularity of these tissues (Figure 3, B and C, and Supplemental Figure 4, A–C). Tumor regions in phenotype cluster 1 are mainly comprised of genotype cluster 1 and mutation cluster 2, indicating an intermediate level of singly amplified *EGFR* and *CDK4* cells, with a slightly higher frequency of *TERT*p-mutant cells compared with phenotype cluster 0 (Figure 3, B and C, and Supplemental Figure 4, A–C). Given the high cellularity of phenotype cluster 0, it is plausible that the majority of these tumor regions are enriched in cancer cells harboring genetic changes not captured by our probes. Cells with *EGFR*-only amplification were depleted from regions rich in hypoxic cells, which was not observed for *EGFR*/*CDK4* co-amplified cells (Figure 3, D and E). This is in line with the previously reported angiogenic role of amplified EGFR signaling (10).

We also noted that the connections between genotype- and phenotype-based classifications change between primary and recurrent tumors (Figure 3B). In recurrent tumors, genotype cluster 3 environments, with high frequencies of both *EGFR*-only amplified and *EGFR*/*CDK4* co-amplified cells, lose their connection with hypoxic (phenotype cluster 4) and tumor-cell-rich nonvascularized immunodepleted environments (phenotype cluster 5). We instead observed a transition of genotype cluster 3 environments to tumor-cell-rich neighborhoods, with increased frequency of EC mimicry and intermediate levels of hypoxia and immune infiltration (phenotype cluster 2). Genotype cluster 2, rich in *CDK4*-amplified cells, associated with hypoxic and immune-rich environments in primary tumors switched to a tumor-enriched environment in recurrent tumors. These frequency-based analyses confirm that genomic amplification may constrict the evolvability of GBM cells and may impact the adaptation of the TME upon recurrence.

Since intercellular interactions depend on cellular proximity to their neighbors, we next performed clustering analysis considering the spatial localization of the cells analyzed in this study. We built a distance matrix and neighborhood feature vectors based on the Euclidian distances from each cell to its nearest neighbors for each tumor region analyzed in our study. Comparison of proximity-based clustering shows that spatial arrangement preferences for immune cells and vascular cells are comparable between primary and recurrent tumors (Figure 3F). However, *CDK4* single-amplified cells have more genetically diverse neighbors in recurrent tumors than in primary tumors. Given that hypoxia is reported as contributing to the processes of EC mimicry and vasculogenic transformation, it was interesting to observe hypoxic EC-mimicking cells that were less abundant in primary tumors overall, possessing a higher diversity of neighbors in this setting. In recurrent tumors, hypoxic EC-mimicking cells increase in number but are primarily in proximity to other tumor cells and especially those with *TERT*p mutation. Hypoxia levels increasing over the course of disease may lead to an increased adaptation of malignant cells by adopting the EC phenotype. Initial stochasticity of this process could result in a more dispersed spatial arrangement of the EC-mimicking cells within the TME.

In summary, our results demonstrate that a significant shift in cellular composition from primary to recurrent GBM is reflected in single-cell genetic heterogeneity, but is also linked to tumor cells’ ability to adopt an EC/pericyte-like phenotype. This cellular adaptation is associated with local hypoxia and co-occurrence of all 3 oncogenic amplifications driving the distinct cellular states in GBM (2). It is plausible that the high level of aneuploidy does not allow these cells to thrive in a heterogeneous tumor, but provides a selective advantage under the pressure of chemotherapy and radiation.

### Texture of ECM revealed by reflectance imaging associated with genetic diversity.

**Figure 4:**
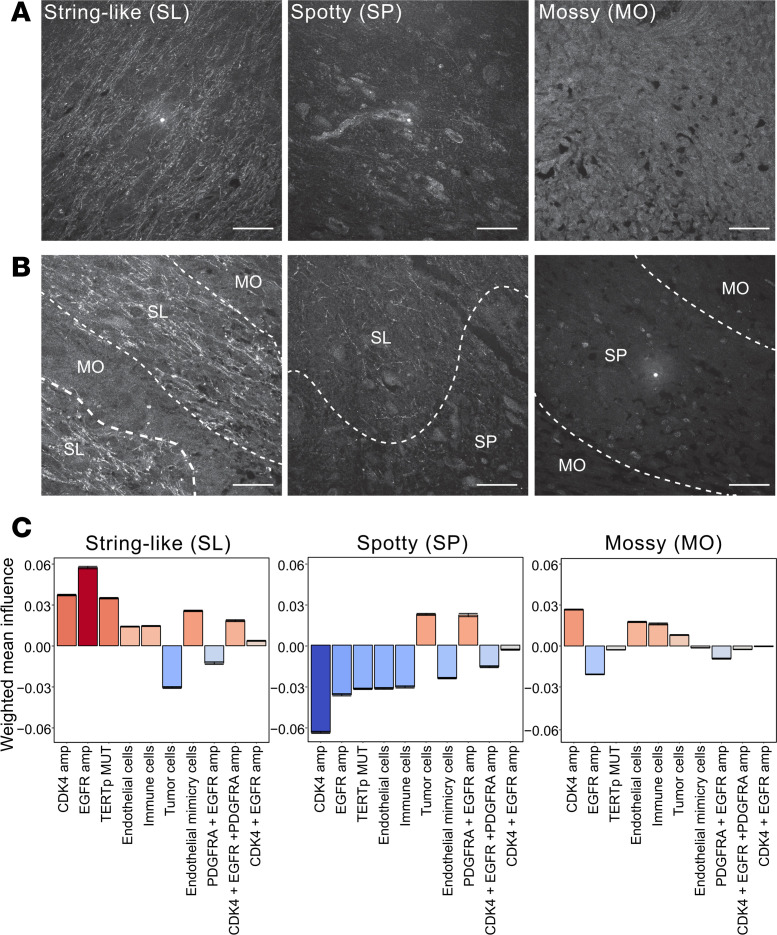
Reflectance imaging–based patterns of tissue organization predictive of cellular composition of the tumor. (**A**) Representative images of reflectance microscopy–based extracellular matrix textures: string-like, spotty, and mossy. Scale bars: 40 μm. (**B**) Representative images of mixed reflectance textures and their manual annotation. SL, string-like texture; SP, spotty texture; MO, mossy texture. Scale bars: 40 μm. (**C**) Influence of cell type on reflectance niche. Mean non-zero influences estimated from correctly classified quadrats (quadrat width and height = 199), weighted by the frequency at which each feature had a non-zero influence per class. Error bars indicate the 95% confidence intervals. *n* reps = 100, mean correctly classified quadrats per rep = 690.49, median ROC-AUC = 0.784.

The differential composition of the ECM, with elevated levels of collagens, laminins, and hyaluronan, is another factor contributing to local microenvironment heterogeneity within GBM tissue (11). To image ECM structures without the need to add additional fluorophores to our staining panel, we took advantage of the differential refractive index of ECM components, which results in the scattering of light off the ECM structures generating a label-free contrast image by reflectance confocal microscopy (RCM) (12). Thus, we included RCM imaging to visualize the organization of the ECM in the first round of our imaging protocol. Across 90 imaged tumor areas, we identified 3 classes of ECM texture: “string-like,” “spotty,” and “mossy” (Figure 4A), with a majority of the fields of view (FOVs) containing a mixed texture (Figure 4B and Supplemental Table 3).

Species distribution models (SDMs) are an ecological method to measure how environmental factors and species are spatially associated with a niche of interest and have previously been used to study the TME (13). We thus developed a neural network–based SDM to better understand the spatial relationship between cellular composition and the local ECM texture. The manual annotation of ECM texture was used to perform quadrat counting on each FOV’s cell segmentation data (see Methods for details). The network was then trained to predict a quadrat’s ECM texture given the abundance of each cell type in that quadrat. After training, the relationship between the abundance of each cell type and the ECM was quantified using the Integrated Gradients feature attribution method (14). This approach allows for the exclusion of incorrectly classified quadrats, meaning that the estimates of the relationship between cell types and the ECM are only based on correct classifications. After applying the Integrated Gradients method to 100 trained models, each cell type’s average attribution was calculated and multiplied by the frequency with which that cell type had an informative attribution (i.e., associated with a correct classification and had non-zero attribution; Supplemental Figure 5). The weighted non-zero attributions for each ECM texture show significantly different contributions from different cell types (Figure 4C). String-like texture is strongly associated with the presence of *CDK4*-amplified cells, *EGFR*-amplified cells, and *TERT*p-mutant cells, yet not associated with overall frequency of tumor cells (all cells with amplifications or mutations). In contrast, spotty ECM texture is linked to higher overall frequency of tumor cells and mildly related to presence of cells with co-amplified *PDGFRA/EGFR*. These results suggest that cells with *PDGFRA/EGFR* co-amplification may have distinct microenvironmental niche preferences compared with cells with other genotypes identified in this study. Thus, despite its heterogeneous origin, we show that reflectance can operate as a meaningful tool to classify TMEs.

### Reflectance and spatial transcriptomics identify a cluster of perivascular immunosuppressive macrophages.

**Figure 5:**
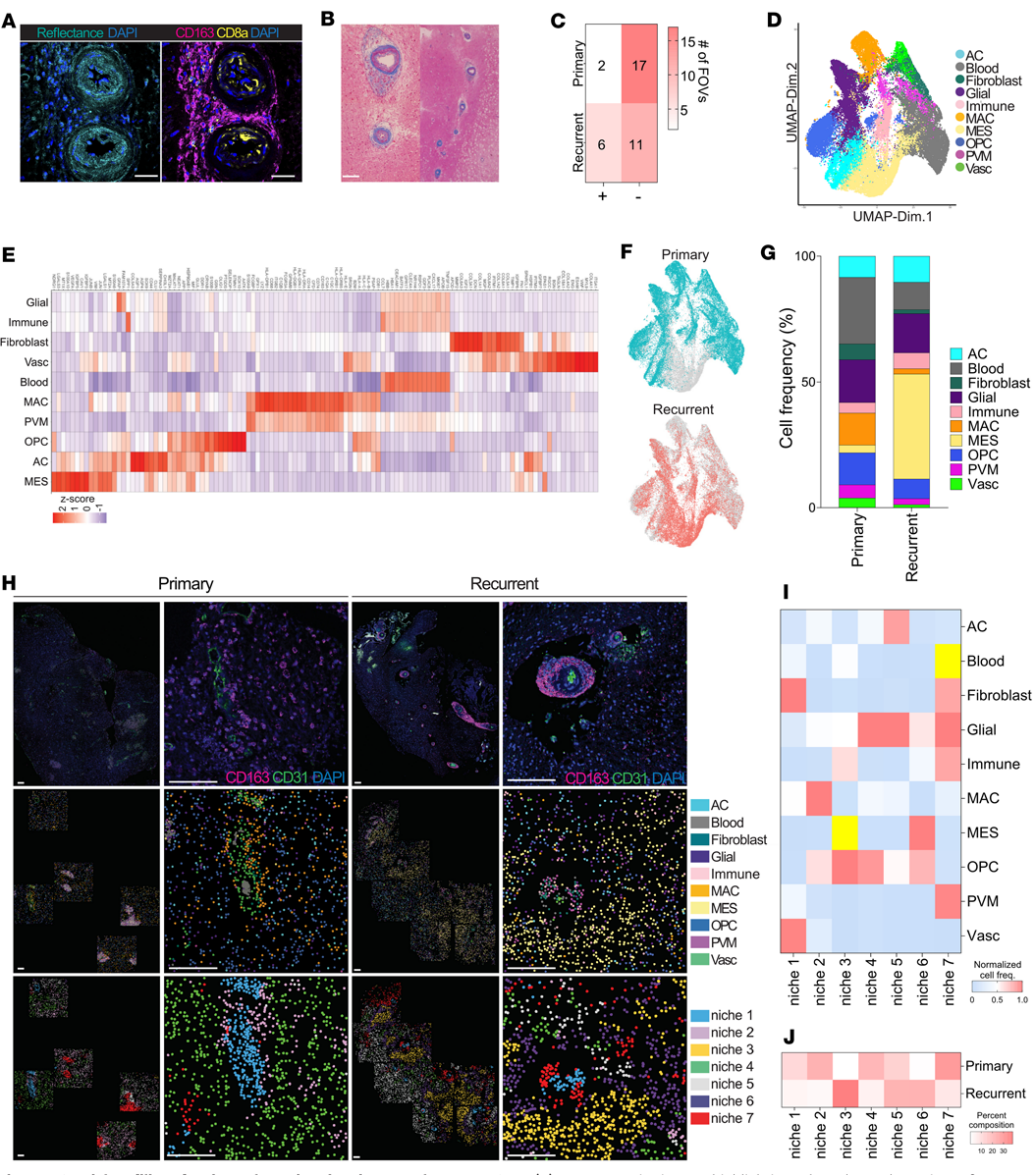
Spatial profiling of perivascular regions in primary and recurrent GBM. (**A**) Representative images highlighting selected vascular regions of recurrent GBM samples. Reflectance imaging showing hyperintense signal around vessel lumen (left) and immunostaining for CD163 and CD8α of peripheral cells (right). Scale bars: 40 μm. (**B**) Trichrome staining of perivascular collagen in recurrent GBM of adjacent section of the tumor region imaged in panel **A**. Scale bar: 40 μm. (**C**) Quantification of collagen rim around blood vessels in FOVs containing vasculature for primary and recurrent samples. Color scale represents number of cases. (**D**) Semisupervised clustering generated from normalized gene expression of 52,588 cells (4 GBM tumors). (**E**) Top differentially expressed markers of cellular groups. Columns represent cell types and rows represent genes. Scaled expression data represented as *z* scores. (**F**) UMAP plot grouped by tissue of origin. Primary (top) or recurrent (bottom) tissues. (**G**) Cell type composition of primary and recurrent samples. (**H**) Representative images of linked IHC (top), cell type spatial plots (middle), and niche spatial plots (bottom) in primary and recurrent samples. IHC images represent matched tissue sample locations to spatial plots at serial section not more than 12 μm away. Scale bars: 120 μm. (**I**) Distribution of percentage of cell types present within TME niches. Yellow cells indicate highly represented outliers computed at α = 0.001 before normalization. (**J**) Niche composition of primary and recurrent tumor.

While classifying the reflectance images, we noted that the majority of the blood vessels in recurrent tumors are surrounded by a thick layer of ECM deposition (Figure 5, A–C). Vascular malformations, including vessel hyalinization, are frequent side effects of radiotherapy (15). Trichrome staining confirmed that these structures are highly enriched in collagen (Figure 5B). Interestingly, immunofluorescent staining revealed that the perivascular collagen rims are densely populated by immune cells, and that a vast majority of these cells are CD163^+^ (Figure 5A). CD163 is a marker of immunosuppressive polarization of tumor-associated macrophages, previously linked to GBM survival (16). The tight localization of these cells and protein around blood vessels possibly impede other immune players like CD8^+^ cells (Figure 5A) and hematogenous components from exiting the vasculature and could also have a supporting function for blood vessel structure.

To test these hypotheses and elucidate the functional role of the perivascular immunosuppressive macrophages in recurrent GBM, we performed single-cell spatial transcriptomic characterization of 2 matched primary and recurrent human GBM samples using the CosMx platform (17). A total of 36 regions of interest containing blood vessels and surrounding tissue were selected (Supplemental Figure 6). CosMx enabled localization of 1,030 transcripts at single-cell resolution by performing multiple cycles of nucleic acid hybridization of fluorescent molecular barcodes on FFPE tissue. Our analysis was performed on 52,588 cells, with an average of 106 transcripts detected per cell (Supplemental Tables 4 and 5). Semisupervised Leiden clustering of both primary and recurrent samples identified 10 major cellular clusters, annotated based on established markers of cell type metaprograms (2) (Figure 5, D and E). Recurrent samples were enriched in mesenchymal-like tumor cells (“MES”), expressing high levels of NDRG1 and VEGFA that further distinguish them as hypoxic (Figure 5, F and G, and Supplemental Figure 7). We observed these MES cells arranging in spatial niches surrounding collagen-rimmed vessels (Figure 5H).

Of note, 2 different types of immune cells were associated with the blood vessels in recurrent tumor tissue: the perivascular macrophages (“PVM”), expressing CD68, CD14, CD74, and CD163, corresponding with CD163^+^ immunostaining, and the SPP1-, CCL7-, and CEACAM1-expressing cells (“Immune”) — most likely tumor-infiltrating lymphocytes, which would correspond to the spatial positioning of CD8a^+^ immunostaining (Figure 5, A, E, and H). While PVM cells do not appear to be the major source of collagen in the perivascular spaces (Supplemental Figure 7), they are in closest proximity to the fibroblast-like cell type that highly expresses COL1A1, COL3A1, and COL6A3. Notably, this fibroblast-like cell group also highly expresses DCN, encoding decorin, an ECM proteoglycan that binds collagen (Figure 5E).

Next, we performed niche analysis combining all the profiled tissue areas. Seven niches were identified, with variable abundance in primary and recurrent samples (Figure 5, H–J). The most interesting was the organization of the niches surrounding the blood vessels in recurrent tumors. In these areas, niche 1, which is a vascular niche composed of ECs and fibroblasts, was surrounded by niche 7, enriched with immune cells, glial cells, and fibroblasts (Figure 5I). PVM cells of recurrent tumors were primarily found in this zone. These areas were also encapsulated by niches rich in mesenchymal and OPC-like cells (Figure 5H). No such structured organization was observed around vasculature of primary tumor samples. Rather, niche 2, which is enriched with a different macrophage cell type (“MAC”) was observed to be more localized around vasculature and widely distributed in the TME of primary tumor samples (Figure 5, H–J). Surprisingly, niche 7, associated with perivascular regions in recurrence, was only identified in primary tumor areas possessing aggregated blood cells and PVM surrounded by glial cells, suggesting ruptured vessels with hemorrhage and glial scarring (18) (Figure 5H).

**Figure 6:**
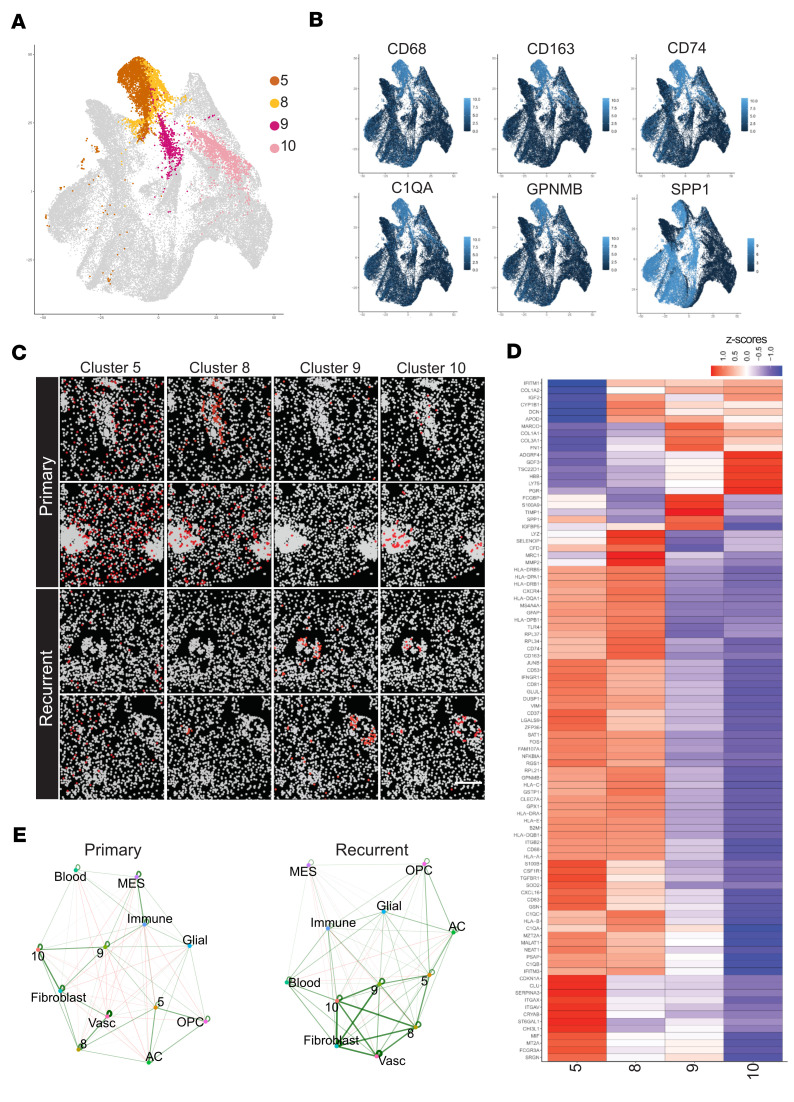
Perivascular niche macrophage cellular interactions in vessels with or without collagen rim. (**A**) UMAP plot highlighting cell type clusters 5, 8, 9, and 10 (higher resolution clustering of previous MAC and PVM cell type clusters). (**B**) Feature plots depicting macrophage/monocyte-derived cell type gene expression markers enriched in the areas of cluster 5, 8, 9, and 10. (**C**) Spatial plots of cell types from cluster 5, 8, 9, and 10 in primary and recurrent GBM. FOVs chosen possess vasculature validated through IHC and gene expression. Scale bar: 120 μm. (**D**) Top differentially expressed markers between clusters. Columns represent cell types and rows are genes. Scaled expression data represented as *z* scores. (**E**) Cell-cell interactions in primary and recurrent tumor tissue. Green lines show spatial proximity enrichment and red lines show depletion between pairs of cell types. Proximity enrichment derived by calculating the observed over the expected frequency of cell-cell proximity interactions. The expected frequency is the average frequency calculated from the spatial network simulations.

Since our initial unsupervised analysis identified 4 clusters of macrophages, 2 of which seemed to have the PVM phenotype, we asked whether this cluster could be further resolved to differentiate true perivascular macrophages from the ones linked to hemorrhagic areas in the tissue. Indeed, we found that these 4 clusters correspond to 4 spatially distinct locations (Figure 6, A–D; reanalysis of data presented in Figure 5). Cluster 5 macrophages are widely distributed in the tissue (Figure 6C). Clusters 8 and 9 are both associated with vasculature, but only cluster 9 cells are found next to vessels with collagenous deposition in recurrent samples (Figure 6C). Interestingly, cluster 9 cells express significantly higher levels of collagen-encoding COL1A1 and COL3A1, as well as fibronectin (FN1) and thus may contribute to the thickening of the perivascular ECM (Figure 6D). Cluster 10 macrophages are predominantly residing within the hemorrhagic areas (Figure 6C). The differences in perivascular niche composition between primary and recurrent tumor were also evident in cell-cell proximity analysis (Figure 6E). In primary tumor, ECs interact directly with fibroblasts and macrophage cluster 8, while in recurrent tumor there is enrichment of interactions between the ECs, fibroblasts, and macrophage clusters 8, 9, and 10. Thus, while transcript-based identification of distinct phenotypes of macrophages might be challenging with non–genome-wide spatial transcriptomics, the analysis of the localization of suspected cell types yields clear spatial differentiation between primary and recurrent GBM.

## Discussion

The presence of genetically distinct clones within different regions of GBM tumors is evident on multiple levels, from bulk analysis of multiple regions of the same tumor by sequencing (19, 20) or FISH studies (21) to inference of copy number from single-cell transcriptomics (2). The spatial context of the TME and its relationship with the clonal evolution of the tumor is still an emerging field. Our study provides direct insight into the link between genetic drivers of specific GBM cell states and microenvironmental factors, including hypoxia and immune predation, in matched primary and recurrent samples.

Recently, 2 studies have gleaned into the spatial heterogeneity of GBM and identified hypoxia as one of the major factors influencing transcriptional cell states within the tumor tissue (3, 4). Spatial gradient of hypoxia results in a concentric organization of cellular states with mesenchymal features surrounded by hypoxia-responsive cell states and vasculature. This is similar to our observation of mesenchymal-like cells in the vicinity of blood vessels in recurrent GBM, although in our data set the niche-organizing factor seems to be the underlying vascular structure, presumably with abnormal functionality. Our comparison of matched primary and recurrent GBM revealed an increase in hypoxic EC mimicry in recurrent disease, another vasculature-related anomaly driving disease progression. GBM cells’ ability to adopt an EC- and pericyte-like phenotype in recurrence has been previously shown both in model systems and in human tumor tissues (22, 23), and has been linked to radiation-induced vascular differentiation (9). While contradictory observations fueled the debate of the tumor cell-of-origin of ECs, our study classified EC mimicry based on presence of DNA amplification (>6 copies) and CD31 expression. Thus, we confirm that EC mimicry is one of the key phenotypic changes in human recurrent GBM, likely fueled by radiation-induced hypoxia.

Another finding specific to blood vessels in recurrent tumors in our study, enabled by reflectance imaging, was the identification of the structured collagen rim surrounding the blood vessels in recurrence. Within these structures, we uncovered interactions between immunosuppressive macrophages and fibroblast-like cells secreting decorin. Decorin is a small leucine-rich proteoglycan and a transforming growth factor β (TGF-β) antagonist, implicated in a variety of pathophysiological processes, such as collagen fibrillogenesis and wound healing (24). It also functions as a tumor suppressor by directly binding to receptor tyrosine kinases, including VEGFR2, EGFR, PDGFR, and MET, and triggering catabolic and antiangiogenic programs in tumor cells (24). In mouse and rat models of glioma, decorin was shown to inhibit tumor growth by regulating the immune response mediated by T cells and microglia (25, 26). M1-polarized macrophage autophagy and an increase in M2-polarized macrophage numbers have also been shown as an effect of decorin-mediated Toll-like receptor (TLR) signaling, albeit in the context of tubular kidney damage (27). Thus, decorin could play a key role during instances of vascular injury and inflammation resulting from disruptions in the blood-brain barrier and aberrant angiogenesis in GBM. Secretion of decorin in response to vascular injury by vessel-associated fibroblast-like cells specific to recurrent tumors could be controlling the polarization of macrophages and orchestrating the resolution of the inflammation at these sites. Perivascular decorin acting on collagen and other components of ECM may also decrease the local tissue stiffness and facilitate extravasation of the bone marrow–derived macrophages. Modulation of microenvironmental stiffness alone can also affect metabolism, proliferation, and migration of GBM cells (28). It is possible that collagen formation around the ECs is a mechanism to fortify vasculature and resist full-fledged collapse. The resulting inflammation and fibroses attract injury associated macrophages with M2 phenotype for wound healing and antiinflammation. The trafficking of macrophages expressing collagen and fibronectin to these perivascular sites would further thicken the vascular barrier and contribute to the metabolic zonation of nearby tumor cells by modifying the gradient of exposure to oxygen, nutrients, and paracrine signals (29). Whether surrounding tumor cells underwent a hypoxic transformation or preferentially sought out these decorin-influenced perivascular spaces has yet to be determined. The positioning of “mesenchymal-like” cancer cells at a certain distance from the cells expressing decorin may allow them to escape its antiproliferative effects. The prognosis of “mesenchymal-like” tumors are the worst among all GBM transcriptomic subtypes, and these hypoxia/perivascular associations could be exploited to understand their aggressive behavior. Unique sensitivities of GBM cells within the perivascular niche will be an ongoing area of investigation. Understanding the molecular and cellular responses within this niche is crucial for devising targeted therapeutic strategies tailored to the specific challenges posed by the vascular abnormalities associated with GBM and its current treatment.

In addition to characterizing unique perivascular niches based on ECM features in recurrent tumors, label-free reflectance microscopy also allowed us to correlate the texture of the ECM with genetic and phenotypic traits. Notably, this texture of the tumor tissue emerged as a potential marker for spatial heterogeneity related to clonal selection. The string-like appearance of the ECM could be attributed to the highly reflective myelination of white matter neuronal axon tracts. Interactions of GBM cells with neurons stimulate proliferation of cancer cells and provide tracts for their invasion into the brain parenchyma (30). Thus, it is likely that neuron- or neuronal axon–rich environments provide yet another niche selecting for GBM cells with particular traits. Our results suggest that *TERT*p-mutant cells and *EGFR*-amplified cells may favor this environment or perhaps contribute to its inception. The physical properties of ECM organization have also been shown to promote a more mesenchymal phenotype of GBM cells (31). To unravel the full extent of the hidden structure within the ECM, future studies should include proteomic analyses to provide a more comprehensive understanding of the molecular composition of the ECM and cell types associated with these unique structures. Given that GBM cells are known to differentiate into neuronal and neuroglia-like cellular states, a more in-depth proteomic elucidation alongside genomic studies could shed light on potential molecular interactions and cellular functions crucial for ECM niche establishment.

Comparing matched primary and recurrent GBM characteristics has been historically hampered by relatively low rates of recurrent brain tumor resection. Multicenter consortia-based collaborations enabled genomic and transcriptomic studies at a larger scale, uncovering increased clonal diversity (20), higher frequency of mesenchymal phenotype (32), and highlighting the importance of reorganization of TME and ECM in recurrent disease (33). Despite the limited number of cases presented in this study, the in-depth analysis of single-cell spatial heterogeneity points to specific niches and unique cell interactions that can be further explored with larger cohorts.

Our study demonstrates that the spatial localization of macrophages is an important dimension providing context for identification of distinct subpopulations of these cells. Several single-cell studies demonstrate competition and specialization of these cell within the brain tumor tissue (34, 35). Moreover, cancer-associated fibroblasts have been recently linked to M2 polarization of macrophages in GBM (36). Our results show that this interaction may be unique to perivascular niches and may contribute to increased immunosuppression in recurrent GBM. New technologies allowing for whole-transcriptome single-cell spatial interrogation in archival tissues will provide a more detailed view of receptor-ligand pair interactions to directly tie cellular states and functions within distinct niches of GBM.

## Methods

## Sex as a biological variable

Human tumor samples in this study include both sexes. The details are shown in Supplemental Table 1. Due to the limited cohort size, the study was not powered to analyze the effect of sex.

### Human tissue samples

GBM pathology was confirmed for each FFPE block by a board-certified neuropathologist. The cohort was comprised of 9 cases of matched primary and recurrent IDH-WT GBM. Case 10 was added for spatial profiling and also consists of matched primary and recurrent IDH-WT GBM. Clinical details are shown in Supplemental Table 1.

### IF staining

After deparaffinization and rehydration, slides were subjected to antigen retrieval in citrate buffer (pH 6; Dako) for 12 minutes in a steamer. Blocking solution (10% goat serum in PBST) was applied for 30 minutes. Incubation with primary antibody in PBS was held overnight at 4°C in a moist chamber. Secondary antibody was applied for 1 hour at room temperature. Samples were mounted with Slowfade Glass Mounting Medium with DAPI (Vector Laboratories). The antibodies used are listed in Supplemental Table 6. Imaging was performed on an Olympus FV3000 confocal microscope.

### FISH and STAR-FISH

Bacterial artificial chromosome clones RP11-339F13 (*EGFR* gene), RP11-571M6 (*CDK4* gene), and RP11-231C18 (*PDGFRA* gene), were obtained from BACPAC Genomics and validated by PCR and fluorescence in situ hybridization (FISH) on xenografts with known amplification status (5). FISH probes were generated by nick translation (Abbott Molecular, 07J00-001) using fluorescent dUTPs (Abbott Molecular). STAR-FISH primers and probes for the *TERT*p C228T mutation were purchased from IDT and Life Technologies (sequences as previously described; ref. 5).

For multiplexed immunofluorescence-FISH (IF-FISH), the FFPE slides were first stained as described in *IF staining*. Imaging with resonant scanner on Olympus FV3000 confocal microscope was used to create a map of the tissue. Five regions of interest were selected at random in each specimen, with user guidance to reject areas with poor tissue quality. High-resolution images of regions of interest were then collected with HD detectors and the XY image coordinates were saved, to enable automated stage repositioning and imaging of the same area after FISH staining.

Subsequently, the imaged slides were demounted, quenched with 0.005% pepsin in HCl at 37°C, washed, and prepared for STAR-FISH, as previously described (5, 7). STAR-FISH allows for in situ point mutation detection at the single-cell level. Briefly, permeabilized FFPE slides were subjected to 2 rounds of in situ PCR with point mutation–specific primers. The first round of PCR amplifies genomic DNA region of interest and the second allows for addition of unique overhangs to mutation-specific amplicon. Next, a fluorescently labeled probe was hybridized to the unique overhangs on the amplicon. The distinct spot-like signal of STAR-FISH was then detected in the nuclei of the mutant cells within the tissue by confocal microscopy.

Post–STAR-FISH images were acquired in *Z*-stacks (average of 5 slices per image) on an Olympus FV3000 confocal microscope, with XY position matching the IF image. The DAPI channel was used to manually correct the localization. All images were acquired in sequential mode to avoid fluorophore cross-talk.

### Spatial transcriptomic profiling

Spatial transcriptomic profiling was performed with a CosMx Human Universal Cell Characterization RNA Panel (1000-plex) plus 30-plex GBM panel (Nanostring) (17) on 4 FFPE samples: 2 biopsied regions from a primary GBM and 2 biopsied regions from a matched recurrent for a total of 36 FOVs (*n* = 22 across 2 primary tumor regions and *n* = 14 across 2 matched recurrent tumor regions). FOVs for transcriptomic analysis were selected based on whole-tissue immunofluorescence imaging of CD163, CD31, GFAP, and DAPI and matched with the morphology visualization panel (B2M/CD298, GFAP) on CosMx Spatial Molecular Imager (SMI). A total of 52,588 cells were identified, with an average of 111 and 99 transcripts per cell in primary and recurrent samples, respectively (Supplemental Tables 4 and 5).

### Quantification and statistical analysis

Statistical details for each experiment can be found in the respective figure legends. A *P* value of less than 0.05 was considered significant.

#### Power analysis.

Power analysis with GPower 3.1.9.4 software (https://www.psychologie.hhu.de/arbeitsgruppen/allgemeine-psychologie-und-arbeitspsychologie/gpower) for an a priori sample size utilizing a 2-tailed *t* test was performed to calculate the number of images that should be acquired per slide to compare the presence versus absence of molecular target. Sample size was calculated based on the following study parameters: power of 0.8, significance at 0.05, effect size of 0.2. With each image harboring at least 53 cells, 5 images taken via confocal microscopy adequately power the analysis.

#### Quality control and single-cell genotype analysis.

Image analysis and quantification were performed using a custom analytical pipeline developed in CellProfiler (37) version 4.2.5 designed to detect features of nuclei using automatic thresholding, filtering, and segmentation methods (available at GitHub: https://github.com/mjaniszewska-lab/IF-STAR-FISH).

To ensure only images with preserved of tissue integrity throughout both rounds of staining were analyzed, a quality control step based on the DAPI channel was used. Post–STAR-FISH DAPI image segmentation was used as a mask overlaid on the DAPI image from the first round of imaging to quantify the nuclei that may have been lost in the second round of staining (Supplemental Figure 1). Only 22 nuclei were lost out of total of 20,205 nuclei detected in the first round of imaging.

Intranuclear speckle counting from FISH and STAR-FISH was used to identify nuclei with oncogene (*EGFR*, *CDK4*, *PDGFRA*) amplifications and *TERT*p C228T mutation, respectively. For FISH signal quantification, nuclei were assigned as having a gene amplification in a gene *g* if at least 6 copies of *g* were recorded in that nuclear area, analogous to the *HER2* FISH scoring guidelines of the American Society of Clinical Oncology/College of American Pathologists (38). Coordinates for which 50 or greater copies of any gene were recorded were removed from the data set, as these likely represent overlapping cells. Nuclei with STAR-FISH signal were classified as *TERT*p mutant.

#### Image-based phenotype assignment.

Nuclei were classified as belonging to “immune cell” or “EC” based on their localization within the immunofluorescence signal segmentation region positive for CD45RO or CD31, respectively. Segmentation was performed using custom analytical pipeline developed in CellProfiler (37) version 4.2.5 (available at GitHub: https://github.com/mjaniszewska-lab/IF-STAR-FISH). Nuclei with gene amplifications and/or *TERT*p mutation were classified as “tumor cells” based on intranuclear speckle counts. Tumor cells positive for CD31 staining were assigned as “endothelial mimicry tumor cell” class. Nuclei lacking characteristics for all the above-mentioned classifications were classified as “unclassified” based on lack of measured phenotypic features and gene amplifications. Nuclei were also assigned as “hypoxic” using positivity for nuclear HIF1α.

#### Shannon index calculation.

Shannon index of diversity was calculated for every tumor sample using the R *vegan* package (https://cran.r-project.org/web/packages/vegan/index.html).

#### Integrative image analysis.

Using our criteria for image-based phenotype assignment, we performed a series of logic-based operations on count matrices containing CellProfiler information to assign cellular properties to nuclear objects. Genotyping classification specifically categorized, single, double, and triple amplification including equivocal amplification. Phenotyping classification identified different cell types of the microenvironment. Mutation status classification differentiated *TERT*p mutation–positive from *TERT*p mutation–negative cells. We then clustered these cell segmentation data at the cellular level and at the FOV level for both frequency and spatial proximity analysis. To handle cells colocalizing at the exact same point, we added a small random noise to their features in the original matrix and then performed tSNE dimensionality reduction. Finally, we applied multiview clustering of data by pooling cell segments from primary and recurrent labels for differential analysis.

The code written in Python 3.12 related to this analysis is available on GitHub: https://github.com/MathOnco/onubogu_spatial_analysis_of_recurrent_glioblastoma

#### Reflectance quadrat analysis.

Using the principles of species distribution modeling (SDM), we developed a deep neural network (DNN) to quantify the association between each cell phenotype and the local ECM texture, as captured in the reflectance imaging. The inputs to the network were quadrat counts based on the spatial cell segmentation data (described above), and the output the labeled ECM texture (i.e., one of “string-like,” “spotty,” or “mossy”), which could be compared to the ground truth hand annotations.

We performed the model fitting using 4 different quadrat sizes (width and height of 114, 199, 266, or 399 pixels), and found that a quadrat width and height of 199 pixels provided the most accurate classifications. There was a total of 1,270 quadrats, spread across the 90 FOVs. Cell counts were independently scaled between 0 and 1 before creating the train, validation, and test splits, which had proportions of 0.8, 0.1, and 0.1, respectively. The training data set was then balanced by using SMOTE (39) to synthesize minority classes, such that all classes in the training data set had similar frequencies. This process increased the training set from 1016 quadrats to 1793 quadrats. Finally, the training data was split into 11 batches, with approximately 163 quadrats per batch.

The DNN had a “funnel” structure, such that size of the final layers gradually decreased from a maximum width to the number of classes being predicted. More specifically, the complete structure of the network was 12 → 36 (×10) → 24 → 12 → 3, with batch normalization being performed on each layer. To minimize overfitting, dropout layers were also included in the main body of the network (layers 1–11, all of which have 36 hidden units), with the dropout probability decreasing linearly across layers, going from 0.15 to 0. Leaky RELU was used as the activation function for each layer.

Using PyTorch (2.0.1) (40), the above network was trained to minimize the class-balanced focal loss function (41), with ADAMW being used for optimization. To avoid overfitting, we trained the model until the training and validation loss started to diverge, with the former decreasing and the latter increasing. The process of model fitting was then repeated 100 times. This approach resulted in an average of 313.8 epochs per model, with median AUC-ROC scores of 0.66 and 0.784 for the test and overall data sets (excluding SMOTE-synthesized training data), respectively.

The relationship between each input feature (i.e., the cell phenotype) and the network output (i.e., ECM texture classification) was quantified by applying the Integrated Gradients method (14) to each trained network, as implemented in the Python package, Captum (0.6.0) (42). As this method calculates the feature attribution for each sample (i.e., quadrat), we were able to subset our results to include only those quadrats that were correctly classified, meaning we were able to remove inaccurate attributions. This process was repeated on all 100 model fits. Each cell type’s attribution score was then averaged and weighted by the number of times it had a correct non-zero attribution, thus allowing us to account for cases where a feature was most often non-informative (i.e., associated with an incorrect classification and/or had 0 influence on the classification), but very rarely had a high absolute attribution score. By excluding inaccurate attributions, having 100 model replicates to capture more quadrats (i.e., those that may have been correctly classified by one model but not another), and weighting by the frequency of informative attributions, we can have more confidence that the attribution scores are accurate, despite each individual model not having exceptional AUC-ROC scores.

The code related to this analysis is available on GitHub: https://github.com/MathOnco/onubogu_spatial_analysis_of_recurrent_glioblastoma

#### Spatial transcriptomic data analysis.

We used Giotto Suite (4.0.1; https://github.com/drieslab/Giotto/tree/suite) (43) and Seurat (5.0.1; https://github.com/satijalab/seurat) (44) to analyze subcellular transcript information and polygon data generated from the CosMx SMI. A standard Giotto spatial data processing and analysis pipeline was used to visualize spatial clusters. Specifically, raw data from CosMx SMI was loaded into a giotto object. Using polygon centroid coordinates as cell spatial locations, we computed the overlapping feature points to establish an expression matrix onto which we performed data filtering and normalization. A minimum of 5 detected features per cell with an expression threshold of 1 feature were set as filtering parameters. We applied library size normalization, log normalization, and SC Transform normalization (in Seurat) to produce normalized, scaled, and SC transformed normalization values respectively. These features were then used in a typical scRNA-seq workflow, including dimension reduction (PCA and UMAP), creation of a shared nearest-neighbor network, and Leiden clustering to create spatial expression-informed clusters for all FOVs. Expression-informed clustering was used to annotate clusters based on gene expression to establish a list of cell types. To plot differences between the groups we computed candidate marker genes, testing for differential expression among cell types.

Spatially organized gene expression can be analyzed by examining the binarized expression of cells and their spatial neighbors. To investigate the spatial expression patterns in more detail, we created a spatial network based on physical distance of cell centroids and performed binary spatial extraction of genes using the binSpect() function. We used this network to then compute cell-cell interaction enrichment to visualize patterns of cellular positioning. Spatial proximity enrichment or depletion between pairs of cell types was performed using the cellProximityEnrichment() function, which calculated the observed over the expected frequency of cell-cell proximity interactions. The expected frequency is the average frequency calculated from a number of spatial network simulations. Each individual simulation was obtained by reshuffling the cell type labels of each node (cell) in the spatial network.

Using the positional information of each cell, we also computed spatial niches in Seurat. Niche analysis of spatial data distinguishes areas of tissue niches, which are defined by the composition of spatially adjacent cell types (45). The local neighborhood for each cell was determined by the number of spatially proximal neighbors (*k* = 30), counting the number of occurrences of each cell type present in a neighborhood. K-means clustering was then used to group cells that have similar neighborhoods into 7 spatial niches.

### Study approval

All experiments with use of human tumor tissue were approved by Scripps Research IRB protocol IRB-18-7209 and Mayo Clinic and Cleveland Clinic IRB.

### Data availability

Raw image analysis files and analysis code are available on GitHub (https://github.com/mjaniszewska-lab/IF-STAR-FISH and https://github.com/MathOnco/onubogu_spatial_analysis_of_recurrent_glioblastoma). Numerical values for graphs are provided in the Supporting Data Values XLS file.

## Author contributions

UO conducted experiments, analyzed the imaging and spatial transcriptomics data, and wrote the original draft of the manuscript. SP and CDG analyzed the imaging data. KLW and BO helped with image analysis. RS helped with assay design. RV and OS provided clinical samples. ARAA and MJ supervised the study. All authors helped design the study and write the manuscript.

## Supplementary Material

Supplemental data

Supplemental table 1

Supplemental table 2

Supplemental table 3

Supplemental table 4

Supplemental table 5

Supplemental table 6

Supporting data values

## Version 1 05/28/2024

In-Press Preview

## Version 2 06/24/2024

Electronic publication
