# Supplementary material for: Spatial analysis of recurrent glioblastoma reveals perivascular niche organization
Source: JCI Insight. 2024 May 23;9(12):e179853. doi: 10.1172/jci.insight.179853 (PMC11383164; doi:10.1172/jci.insight.179853)
Supplement: Supplemental data [file jciinsight-9-179853-s194.pdf]

SUPPLEMENTAL FIGURES

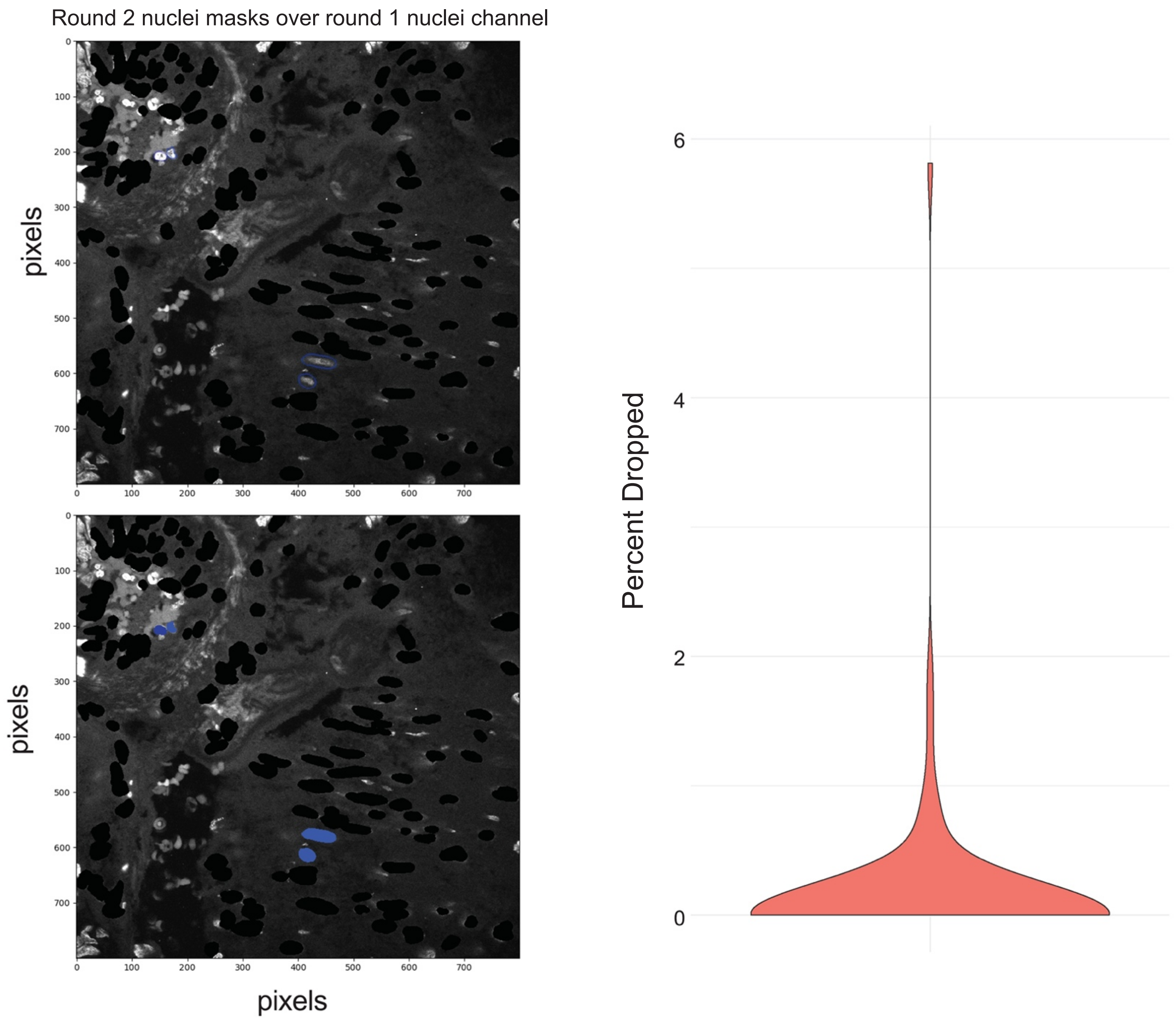

Supplemental Fig. 1. Assessment of nuclei loss after second round on staining and imaging. Nuclear segmentation mask between first and second round imaging. Left panel shows nuclear masks generated from the second round of imaging projected onto the first round DAPI channel. Nuclear objects recognized through masks indicate dropout between first and second round imaging or inaccuracy in automatic segmentation. Right panel shows quantification of nuclei dropout.

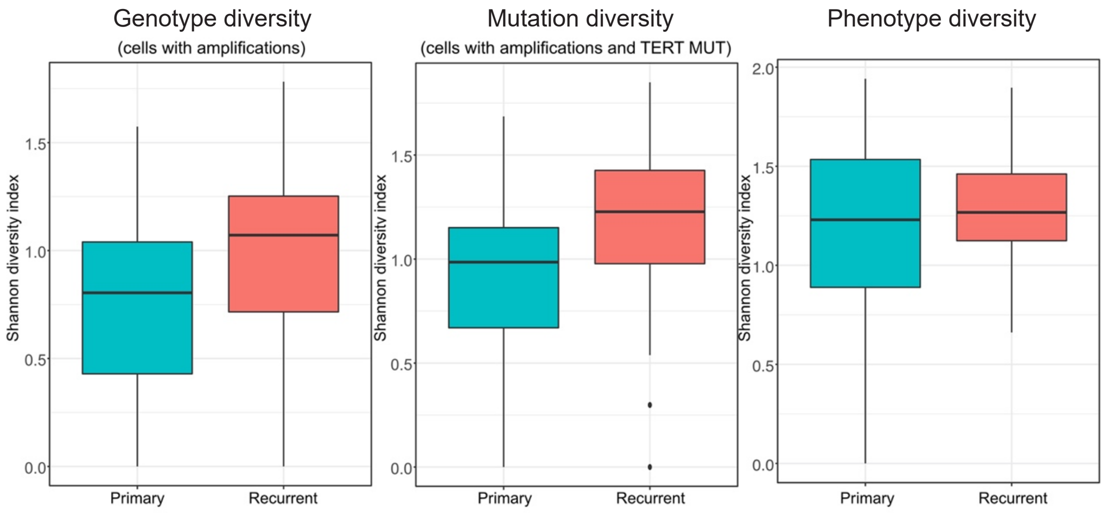

Supplemental Fig. 2. Diversity of genotype and phenotype between primary and recurrent GBM. Shannon diversity index calculated per tumor. The box-and-whisker plots in all bar graphs show the mean (midline) and 25th–75th (box) and 5th–95th (whiskers) percentiles.

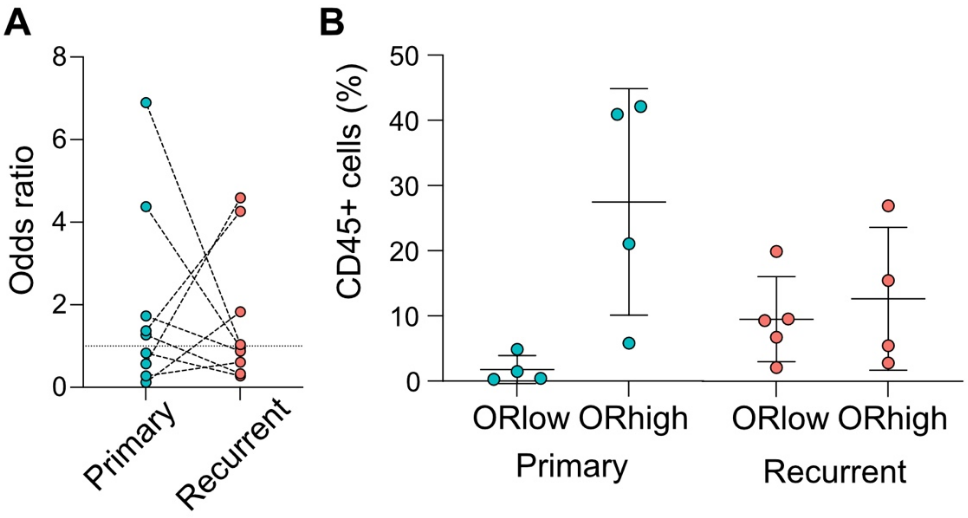

Supplemental Fig. 3. Odds ratio for EGFR and CDK4 co-amplification association with immune infiltration in primary and recurrent tumor samples. A) Odds ratio for EGFR and CDK4 co-amplification in the same cell calculated for each tumor case. Dotted lines connect matching primary and recurrent cases. **B**) Frequency of $CD45^+$ immune cells in tumors classified as OR^{low} or OR^{high} (see Walentynowicz et al. for details). Mean and standard deviation are shown.

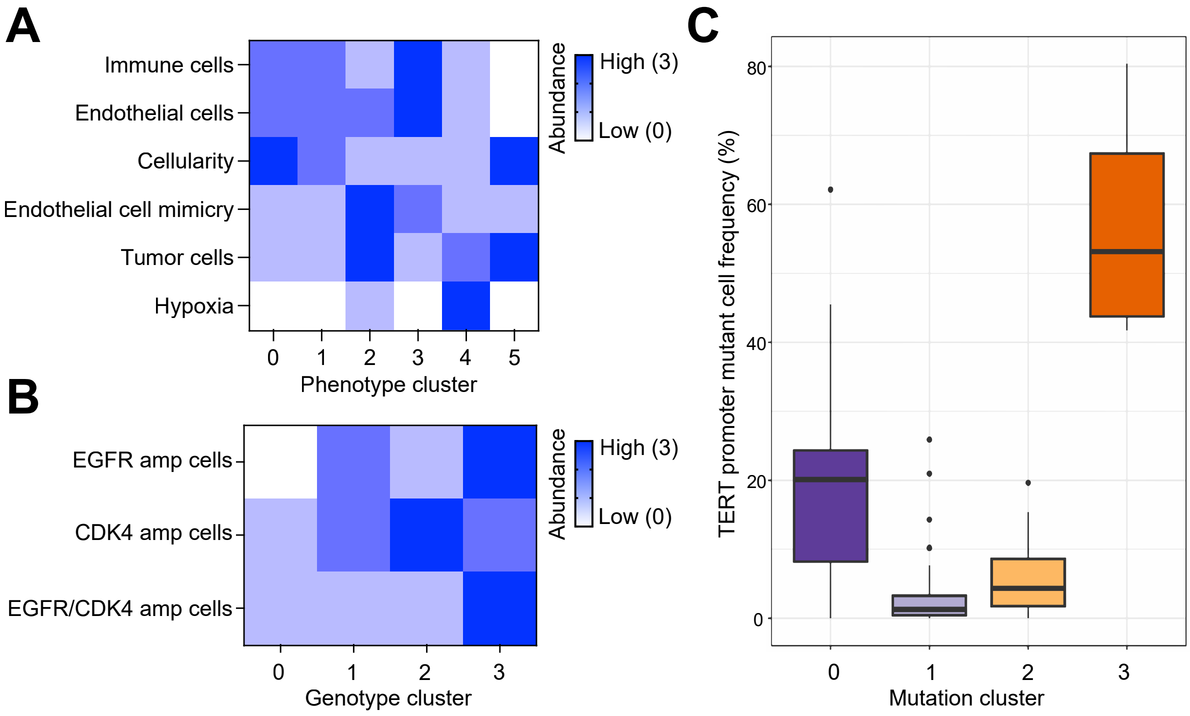

Supplemental Fig. 4. Summary of the phenotype and genotype cluster annotation. The clustering was performed in a semi-supervised manner. The summaries are based on mean abundance across all samples. **A**) Cell type abundance across phenotype clusters. **B**) Genotype abundance across phenotype clusters. **C**) Frequency of cells with TERT promoter mutation across mutation-based clusters. The box-and-whisker plots show the mean (midline) and 25th–75th (box) and 5th–95th (whiskers) percentiles.

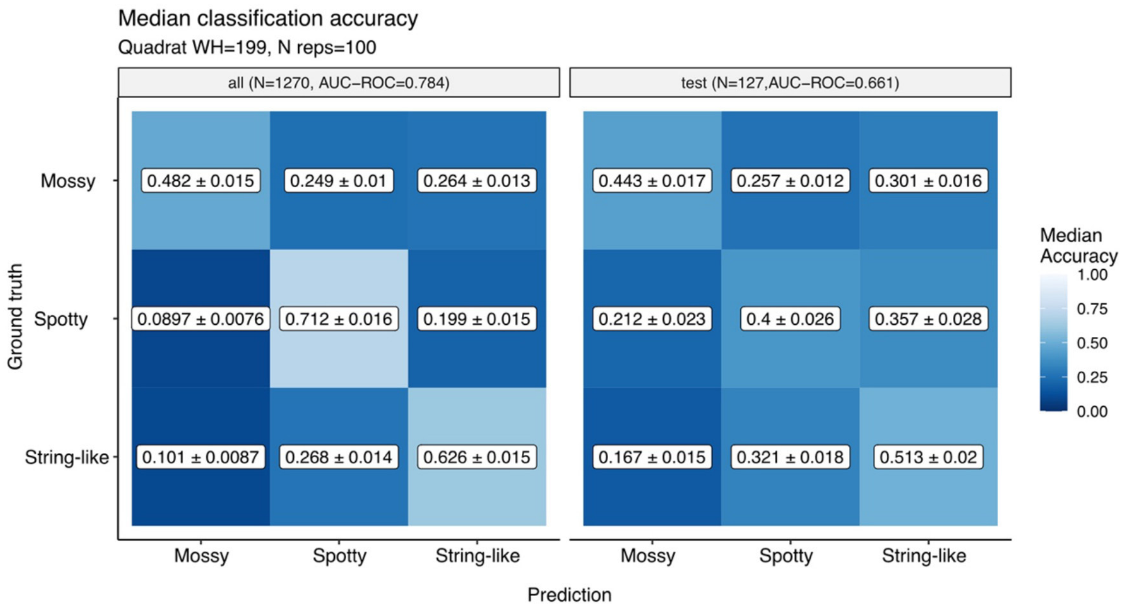

Supplemental Fig. 5. Accuracy of deep neural network classification of reflectance texture. Confusion matrix showing median classification accuracy across 100 runs, plus/minus the margin of error for the 95% confidence intervals. Quadrat WH = 199, N reps = 100.

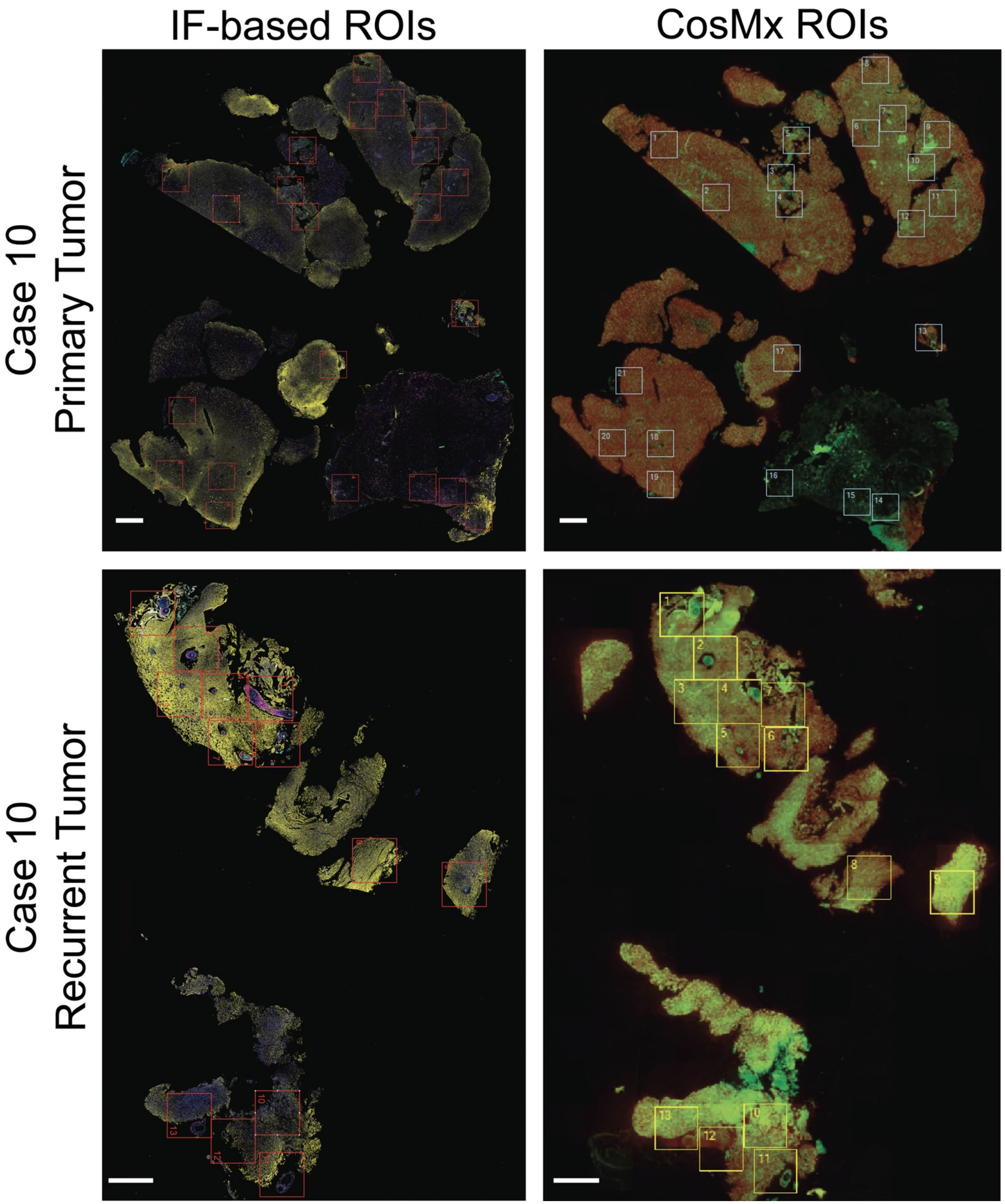

Supplemental Fig. 6. Selection of perivascular areas for spatial transcriptomic profiling. Whole tissue imaging of matched primary and recurrent GBM for CosMX SMI platform. Left panels depict ROIs selected using immunofluorescence imaging of GFAP (yellow), CD31 (green), CD163 (magenta), and DAPI (blue). Right panels show CosMx selected ROIs matched to the same features on 4th or lesser serial section. Scale bar 770 μm.

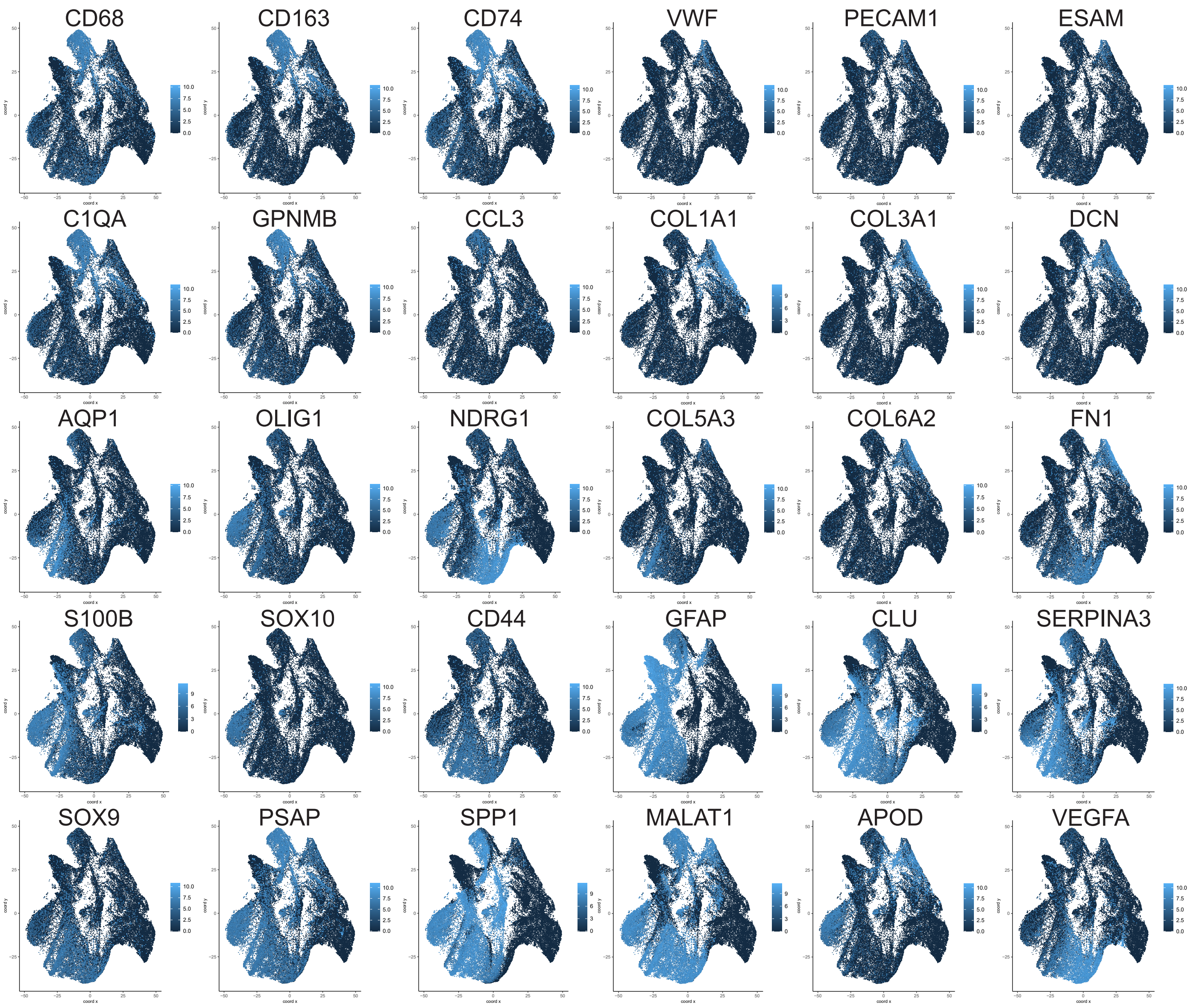

Supplemental Fig. 7. Cell cluster-specific marker expression. Feature plots depicting the distribution of 30 different genes. Selected genes correspond to characteristic marker gene signatures within annotated cell types.

SUPPLEMENTAL TABLE LIST

Supplemental Table 1. Matched primary and recurrent GBM cohort details.

Supplemental Table 2. Single cell classification criteria.

Supplemental Table 3. Frequencies of cells with distinct genotype and phenotype across tumor areas.

Supplemental Table 4. CosMx data quality control summary.

Supplemental Table 5. CosMx 1000plex gene panel details.

Supplemental Table 6. Antibodies used in the study.
